# Care Literacy for Culture, Nature, and Future

**DOI:** 10.12688/openreseurope.17817.1

**Published:** 2024-06-13

**Authors:** Hiroko Costantini, Misato Nihei, Masakazu Sugiyama, Nobuyuki Yagi, James Costantini

**Affiliations:** 1 Oxford Institute of Population Ageing, University of Oxford, Oxford, OX1 4BH, UK; 2 Institute for Future Initiatives, The University of Tokyo, Tokyo, 113-0033, Japan; 3 Graduate School of Information Science and Technology, The University of Tokyo, Tokyo, 113-0033, Japan; 4 Research Center for Advanced Science and Technology, The University of Tokyo, Tokyo, 153-8904, Japan; 5 Graduate School of Agricultural and Life Sciences, The University of Tokyo, Tokyo, 113-0032, Japan; 6 INSEAD Business School, Fontainebleau, 77300, France

**Keywords:** Nature Futures Framework; Nature; Culture; Care Literacy; Sustainability

## Abstract

Within the broader sustainability agenda, an important element relates to the need for a transformative approach to nature. This motivates and is reflected in the Natures Futures Framework. Within this framework, this letter focuses on the relational value of Nature as Culture/One with Nature. This is important yet complex as part of the re-orienting of values to enable truly significant change, and which necessitates individual and community involvement on the value of caring for nature. As a means for understanding and enabling individuals’ potential to engage and contribute, the notion of ‘care for nature literacy’ is put forward.

## Introduction

**Figure 1.**
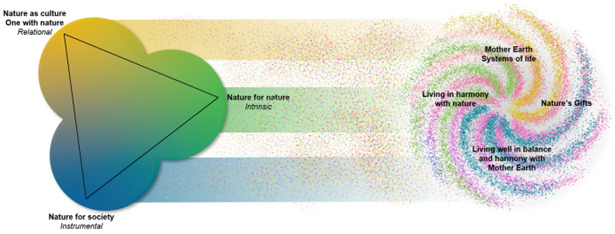
The Nature Futures Framework, with on the left-hand side three value perspectives of nature and on the right-hand side a non-exhaustive representation of knowledge systems and world views on human-nature relationships (Source: IPBES ^4^).

Within the broader sustainability agenda, as per the United Nationals Sustainable Development Goals ^1^, an important element relates to aspects of nature. The extent of challenge to the natural environment is significant, as exemplified by the proportion of planetary boundaries assessed to have been crossed ^2^. The need to shift towards a positive impact on nature is, thus, critical. One important framework for supporting the development of future nature positive approaches is the Intergovernmental Science-Policy Platform on Biodiversity and Ecosystem Services (IPBES) Nature Futures Framework (NFF) ^3^. The framework comprises three perspectives on the value of nature ( Figure 1): Nature for Society, which is primarily an instrumental value; Nature for Nature, which is primarily an intrinsic value; and Nature as Culture/One with Nature, which is primarily relation value ^4^. This letter focuses on the third relational perspective on value, leveraging insights from the first author’s Horizon project leading to the notion of care literacy for an inclusive sustainable society ^5^. The aim of this letter is to put forward an enabler of the behavioural change necessary for the systemic transformation required to achieve nature positive impact.

## Discussion

The complexity underlying the NFF is significant and transparently acknowledged, with one aspect being that across people, societies and cultures there is variation in how the human-nature relationship is conceived (which is represented by the right-hand side of Figure 1). Indeed, the relational dimension has the dual title Nature as Culture/One with Nature ^4^. This is reflected in, for instance, the long running and ongoing challenge of grappling with ‘nature and culture’ in anthropology ^6, 7^. The challenge of addressing the Nature as Culture/One with Nature perspective is also evident in considering specific domains. For instance, in the context of cities and urban planning, important for sustainability, consideration of the NFF highlights how typically used heuristics that aim to guide practice do not generally address the relational value perspective ^8^. Further, tailoring the NFF to application for urban management, one gap highlighted is for indicators to assess the Nature as Culture/One with Nature progress, given the interplay of human and natural systems ^9^. Thus, the challenge in addressing the relational perspective of value of Nature remains even when focusing within specific domains: this would raise the issue that addressing this issue domain-by-domain may only bring partial progress.

Addressing the relational value of Nature as Culture/One with Nature may also be considered at different scales of potential interventions and the interplay across these. The corresponding call for systemic transformation is clear ^10^, as the need for an equitable, just transition ^11^. Based on considering how the NFF perspectives on value may be incorporated into just and inclusive decision processes, system-level change would be complemented by bottom-up agency, with individual and community level engagement to involve also a reflection and potential reassessment of their values ^12^. Considering the relational aspect, this could be viewed as giving more weight to caring for nature ^13^.

Such care for nature would need to be consistent with significant systemic transformation. The connection from people to nature is complex. In tracing through from individual behaviours to impact on nature, there are not only more evident proximate, immediate effects but also indirect, distant and/or future impacts: an important example being the interconnection between consumption choices and sustainability of globally interwoven supply chains ^14^. Further, there is a need for such a system to transform, as motivated the development of the NFF. In such a context, a relevant question is what sort of ‘literacy’ is required to enable people to understand, engage and act; a literacy about their interrelationship with nature.

The idea of an appropriate literacy as being a foundation for people’s active engagement has been applied in several domains, such as for health literacy ^15^. In considering literacy regarding nature, there is a focus on how to educate children, which based on other notions of literacy identifies the need for nature literacy to include motivation, knowledge, competence and confidence to act ^16^. Importantly, this highlights that concepts of literacy not only encompass being able to access information but importantly include being able to infer the implications for oneself, and, crucially, to identify and bring into practice corresponding behaviours. We have put forward “care literacy” ^5^, which has an orientation towards others: whereas, say, health literacy is about own health, care literacy is about care for others. Such care literacy is not limited to enabling care for close ones but importantly also a more generalised notion of care for others in the community. For instance, this broader community scope would include considering how behaviours contribute to inclusion. With its external orientation, care literacy has a parallel with how behaviours may trace through to a potentially broad impact on nature, including proximate as well as indirect, distant impacts.

This points to the potential value of the notion of a literacy about caring for nature, “care for nature literacy”, and developing the corresponding means to assess such a literacy, as is done with other forms of literacy. Such assessments of people’s care for nature literacy would provide one set of indicators to inform understanding at least people’s potential for understanding, engagement and action on the Nature as Culture/One with Nature value perspective. Finally, literacy relating to care for nature would be complementary to literacy for care for others, and thus a means to connect to the overall environmental and social sustainability agenda.

## Conclusion

The need for a transformative approach to nature motivates and is reflected in the Natures Futures Framework. Within this framework, the relational value on Nature as Culture/One with Nature is central, give the need to re-orient values as an enabler of truly significant change. Correspondingly, engagement of individuals and communities on this value of care for nature is important, though a complex domain in which to do so. Development of the notion of ‘care for nature literacy’ would provide a means for understanding and enabling individuals’ potential to engage and contribute.

## Ethics and consent

Ethical approval and consent were not required.

## Disclaimer

The views expressed in this article are those of the authors. Publication in Open Research Europe does not imply endorsement of the European Commission.

## Data Availability

No data are associated with this article.
