# Supplementary material for: Care Literacy for Culture, Nature, and Future
Source: Open Res Eur. 2024 Jun 13;4:115. [Version 1] doi: 10.12688/openreseurope.17817.1 (PMC11411236; doi:10.12688/openreseurope.17817.1)
Supplement: Peer review history

# Reviewer report: Reviewer response for version 1

**DOI:** 10.21956/openreseurope.19261.r43172

**Published:** 2024-09-20

**Authors:** Wessel Ganzevoort

**Affiliations:** 1 Tilburg University, Tilburg, North Brabant, The Netherlands

**Rationale**

 Overall, I enjoyed reading the authors’ letter. The topic is highly pertinent, nicely linking to important contemporary debates on relational values and care for nature, e.g. in the context of IPBES. Furthermore, with their linkage to the notion of care literacy, a notion from studies of health, the authors could make an innovative addition to these debates through their suggestion for a ‘care for nature literacy’.

 However, I do feel that this potential requires a bit of revision of the current version of the letter. As will be detailed below, I feel there are two main issues that could be addressed: the first is that some key terms and arguments could do with stronger explanation or backing in relevant literature, and the second is that the core notion of care (for nature) literary now does not get sufficient attention for the reader to know what next steps could be.

 I hope my suggestions will be of use for the authors, and look forward to seeing their revised contribution.

**Different views**

 The authors drawn on a rich body of literature in their letter, though this also comes with some terminological confusion. See below on my comments related to that, as well as a suggestion for a further theoretical perspective that might be productively drawn on.

**Support by citations**

 Considering the authors’ focus on how to achieve deep structural changes towards differently relating to nature, I feel some core literature is not referenced that might helpfully support the authors in making their argument. Specifically, the literature on leverage points for sustainability transformations seems highly pertinent. Both (Abson DJ, et al., 2017 9Ref 1]) and (Ives CD, et al., 2018 [Ref 2]) have offered in-depth discussions on how to move from surface-level adjustments to deep change in our underlying values and philosophies related to human-nature relationships. I feel drawing on this established literature would be of great help to the authors, as they themselves call attention to systemic transformation and the role of values in paragraph 3. This would also help make paragraph 3 clearer, which is an important building block in the authors’ argument but at present suffers from some vagueness (see comments below).

**Language and terminology**

 I feel there are some important terms that go underexplained in the current manuscript. I recognize that the number of words available to the authors is limited, but at present readers both within and beyond the field are sometimes left wondering how the authors position themselves. For example, the notion of transformation and transformative change is used several times, and it would be helpful for the reader to know what the authors mean by this. Is this identical to the value reorientation discussed, or is one part of the other? And is this a personal or collective transformation, or both? Linking to a definition or explanation of what the authors understand transformation to be of help here.

 The same thing happens in paragraphs 3 and 4 with the notion of ‘care (for nature)’. The authors do cite the work of Jax et al., but without consulting that work it is not clear for the reader what care entails and how it relates to relationality. Considering the fundamentally relation nature of care, explaining this link a bit more could help strengthen the argument the authors make. This is key, since care is fundamental to the recommendations made at the end.

 Note that phrases like “connection from people to nature” don’t actually match well with a relational values perspective, as it describes a one-way relationship whereas relational values point to fundamentally reciprocal relationships.

 In general I feel some more clarity and consistency in terminology would make the message of the paper easier to follow; for example, I wasn’t always clear on the meaning of terms like “aspects of nature”, “challenge to the natural environment”, etc.

**Recommendations**

 I feel at present it is difficult for the reader to know exactly what the ‘theory of change’ of the authors is, because little explanation of the notion of care literacy and how it translates to relational perspectives on nature is given. In the introduction, care literacy is put forward as an ‘enabler of behavioral change’, but how the authors understand this enabling process is not quite clear to me. My suggestion would be to remove current paragraph 2 (which I feel does not add much to the overall argument and currently suffers from significant lack of clarity and relevance), and use this space later in the letter to work through the notion of care literacy and its potential somewhat more.

 Specifically, the reader needs a more explanation of what the authors understand ‘literacy’ to mean. At the end of paragraph 4 they rather suddenly move from systemic change and connection to nature to the question ‘what sort of literacy is required’. The risk here is that literacy can often come with a connotation of knowledge and education, which would point to a very pedagogical perspective on how to inspire relational thinking (e.g. knowledge dissemination and education campaigns). Is that the direction the authors are thinking of here? If so, I feel this could be contested. If the authors dedicate some more words to explaining the notion of care literacy and how it translates to caring for nature, they could make a clearer argument as to what sort of dimensions this includes, and what sort of interventions could strengthen it. I feel that the aforementioned work of Abson et al. and Ives et al. could be very helpful for this (e.g. Abson’s three realms of deep leverage, and Ives’ five categories of nature connections). This would enable the authors to formulate a stronger explanation of what this ‘care for nature literacy’ might look like, how it could be inspired, and how it could then be assessed (as they raise in the final paragraph).

 Having a clearer grasp on what this care for nature literacy looks like would in turn make the link to earlier paragraphs clearer, i.e. how this literacy will support/enable the value reorientation and systemic change called for. It would help clear up unclear sentences like “individual and community involvement on the value of caring for nature”, or for them to “engage and contribute” (to what?). Same thing in paragraph 3 (e.g. ‘individual and community level engagement’(in what?). The final line of paragraph 5 is also rather unclear (e.g. ‘external orientation’). “engage and contribute” is also the final line of the paper; I feel by following up on the recommendations of this review, a stronger final sentence could be written to end the letter on a  highlight.

Where applicable, are recommendations and next steps explained clearly for others to follow? (Please consider whether others in the research community would be able to implement guidelines or recommendations and/or constructively engage in the debate)

No

Does the article adequately reference differing views and opinions?

Partly

Are all factual statements correct, and are statements and arguments made adequately supported by citations?

Partly

Is the rationale for the Open Letter provided in sufficient detail? (Please consider whether existing challenges in the field are outlined clearly and whether the purpose of the letter is explained)

Partly

Is the Open Letter written in accessible language? (Please consider whether all subject-specific terms, concepts and abbreviations are explained)

Partly

Reviewer Expertise:

My research focus is on citizen and community action for nature and sustainability, including citizen science and nature volunteering as forms of action and care for nature. I've also been involved in work on human-nature relationships and relational values.

I confirm that I have read this submission and believe that I have an appropriate level of expertise to confirm that it is of an acceptable scientific standard, however I have significant reservations, as outlined above.

## References

[1] : Leverage points for sustainability transformation. *Ambio* .2017;46(1) : 10.1007/s13280-016-0800-y 30-39 10.1007/s13280-016-0800-y 27344324 PMC5226895

[2] : Reconnecting with nature for sustainability. *Sustain Sci* .2018;13(5) : 10.1007/s11625-018-0542-9 1389-1397 10.1007/s11625-018-0542-9 30220917 PMC6132401

# Reviewer report: Reviewer response for version 1

**DOI:** 10.21956/openreseurope.19261.r42538

**Published:** 2024-09-18

**Authors:** Margherita Paola Poto

**Affiliations:** 1 UiT The Arctic University of Norway, Tromso, Norway

The article under review provides a comprehensive exploration of the Nature Futures Framework (NFF) as outlined by the Intergovernmental Science-Policy Platform on Biodiversity and Ecosystem Services (IPBES). It adeptly discusses the three value perspectives of nature—Nature for Society, Nature for Nature, and Nature as Culture/One with Nature—with a particular focus on the latter. The discussion is enriched by the integration of the Horizon project insights, which introduce the concept of 'care literacy' as a pivotal enabler for behavioral change towards a nature-positive future.

 Detailed Observations

 1. **Framework Complexity and Cultural Dimensions:**

    The article effectively acknowledges the complexity of the NFF, especially in its cultural and relational dimensions. The variation in human-nature relationships across different societies and cultures is well illustrated with references to anthropological challenges (citations 6,7). However, the discussion could benefit from a deeper analysis of how these cultural perspectives can be reconciled or integrated within the NFF. Adding a reference such as [Ref 1] could provide a broader theoretical foundation on care literacy as an integral component of emotional and ecological literacy to enhance understanding across diverse cultural contexts.

 2. **Urban Planning and Indicators:**

    The application of the NFF in urban contexts is discussed, highlighting the lack of indicators for assessing the 'Nature as Culture/One with Nature' perspective. This section is insightful but would benefit from specific examples of proposed indicators or case studies where similar frameworks have been successfully applied. The addition of such details would provide practical guidance for urban planners and policymakers looking to implement the NFF.

 3. **Systemic Transformation and Scale of Interventions:**

    The call for systemic transformation and the need for a just transition are well-articulated. The article discusses the interplay between top-down and bottom-up approaches in fostering systemic change. It would be constructive to include more detailed strategies or models that outline how these scales of intervention could interact more effectively. Further exploration of this interplay could offer valuable insights into operationalizing the NFF at different societal levels.

 4. **Literacy and Engagement:**

    The introduction of 'care literacy' is a novel and compelling concept. The article posits that care literacy could facilitate a deeper connection between individuals and nature, promoting sustainable behaviors. While the discussion on literacy is robust, expanding on how care literacy can be developed, assessed, and integrated into educational and community programs would provide a clearer roadmap for its implementation.

 Constructive Criticism

 The article is well-structured and presents a complex framework in an accessible manner. However, the potential for practical application of the NFF and care literacy could be emphasized more strongly. Providing more concrete examples, tools, or methodologies for applying these concepts would greatly enhance the utility of the article for practitioners and decision-makers.

 Conclusion

 Overall, the article makes a significant contribution to the discourse on biodiversity and ecosystem services by framing the discussion within the NFF and introducing the concept of care literacy. With the suggested enhancements, particularly the inclusion of the reference for emotional and ecological literacy, the article could serve as a seminal piece for academics, practitioners, and policymakers engaged in environmental sustainability and cultural transformation.

Where applicable, are recommendations and next steps explained clearly for others to follow? (Please consider whether others in the research community would be able to implement guidelines or recommendations and/or constructively engage in the debate)

Yes

Does the article adequately reference differing views and opinions?

Yes

Are all factual statements correct, and are statements and arguments made adequately supported by citations?

Yes

Is the rationale for the Open Letter provided in sufficient detail? (Please consider whether existing challenges in the field are outlined clearly and whether the purpose of the letter is explained)

Yes

Is the Open Letter written in accessible language? (Please consider whether all subject-specific terms, concepts and abbreviations are explained)

Yes

Reviewer Expertise:

Ocean Literacy, Emotional and Ecological Literacy, Environmental Law and Governance, Indigenous Law and Indigenous Methodology

I confirm that I have read this submission and believe that I have an appropriate level of expertise to confirm that it is of an acceptable scientific standard.

## References

[1] Emotional and Ecological Literacy for a More Sustainable Society. *https://link.springer.com/book/10.1007/978-3-031-56772-8* .2024;

# Reviewer report: Reviewer response for version 1

**DOI:** 10.21956/openreseurope.19261.r43165

**Published:** 2024-09-03

**Authors:** Maria Huhmarniemi

**Affiliations:** 1 University of Lapland, Rovaniemi, Finland

The complexity of the Nature Futures Framework is rooted in the diverse ways human-nature relationships are understood across cultures and societies. The relational dimension, "Nature as Culture/One with Nature," encapsulates this diversity and poses challenges in urban planning, where conventional practices often overlook relational values. Addressing these values requires systemic transformation and bottom-up engagement, encouraging individuals and communities to reassess their relationship with nature. A proposed "care for nature literacy" could be key to fostering this engagement, emphasizing not only knowledge and competence but also motivation and the ability to act in environmentally and socially responsible ways. Developing means to assess this literacy could provide valuable indicators for understanding and promoting the Nature as Culture/One with Nature perspective, aligning it with broader sustainability goals. - From my own perspective, I would add that arts-based methods could serve as tools for literacy and value transformation.

Where applicable, are recommendations and next steps explained clearly for others to follow? (Please consider whether others in the research community would be able to implement guidelines or recommendations and/or constructively engage in the debate)

Yes

Does the article adequately reference differing views and opinions?

Yes

Are all factual statements correct, and are statements and arguments made adequately supported by citations?

Yes

Is the rationale for the Open Letter provided in sufficient detail? (Please consider whether existing challenges in the field are outlined clearly and whether the purpose of the letter is explained)

Yes

Is the Open Letter written in accessible language? (Please consider whether all subject-specific terms, concepts and abbreviations are explained)

Yes

Reviewer Expertise:

art education, environmental education

I confirm that I have read this submission and believe that I have an appropriate level of expertise to confirm that it is of an acceptable scientific standard.
